# An additional muscle belly of the first lumbrical muscle

**DOI:** 10.1186/1757-1626-1-103

**Published:** 2008-08-18

**Authors:** Soubhagya R Nayak, Ramya Rathan, Rachana Chauhan, Ashwin Krishnamurthy, Latha V Prabhu

**Affiliations:** 1 Department of Anatomy, Centre for Basic Sciences, Kasturba Medical College, Bejai, Mangalore, Karnataka, 575004, India

## Abstract

**Introduction:**

Lumbrical muscles play a vital role in the precision movements of the hand, along with the thenar, hypothenar and interossei muscles. The variation in the lumbrical muscle is clinically significant.

**Case presentation:**

During routine dissection of an adult male cadaver, we observed an additional muscle belly of the first lumbrical muscle took origin from the tendon of the flexor digitorum superficialis (FDS) to the index finger, close to the proximal margin of the flexor retinaculum.

**Conclusion:**

The presence of such an additional muscle in the carpal tunnel should be considered in the aetiology of carpal tunnel syndrome (CTS).

## Introduction

The lumbricals are usually four small muscles of the hand. They are numbered from the lateral to medial side. The lumbricals take origin in the palm from the four tendons of flexor digitorum profundus (FDP), pass distally along the radial side of the corresponding metacarpo-phalangeal joint in front of the deep transverse metacarpal ligament. Each muscle forms a narrow tendon and on reaching the dorsal surface of the proximal phalanx joins the margin of the dorsal digital expansion (DDS) [1]. Lumbrical muscle variation has been reported in the literature by various authors [2-6]. Mehta and Gardner described the anomalous origin of first lumbrical in 2.7% cases they studied [7]. In the present case we observed an additional muscle belly of the first lumbrical muscle took origin from the FDS tendon within the carpal tunnel. The clinical and the phylogenetical significance of the present case have been discussed.

## Case report

**Figure 1:**
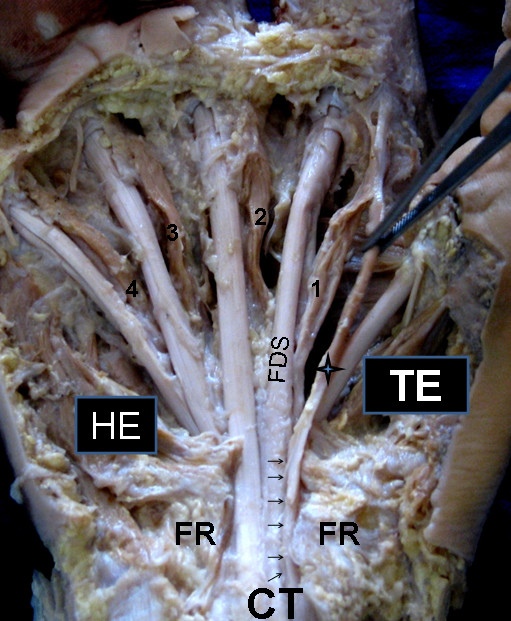
**Right side palmar surface of the hand**. 1, first lumbrical; 2, second lumbrical; 3, third lumbrical; 4, fourth lumbrical; CT, carpal tunnel; FDS, flexor digitorum superficialis tendon to the index finger; FR, flexor retinaculum; HE, hypothenar eminence; TE, thenar eminence. Note the 4-point star indicates the additional muscle belly of the first lumbrical muscle.

During routine dissection of the right upper limb of a 69 year old male cadaver, we observed an accessory muscle belly took origin from the radial side of the FDS tendon to the index finger, at the level of the proximal border of the flexor retinaculum. The accessory muscle belly (71 mm in length & 6 mm in width) was placed lateral to the first lumbrical and joined the same before inserting to the DDS of the index finger. The first lumbrical muscle took origin from the radial side of the FDP tendon for the index finger as expected (Fig 1). Both the first lumbrical muscle and the accessory muscle belly were innervated by a twig from the median nerve.

## Discussion

Lumbricals as a part of the intrinsic musculature is important for its delicate digital movements. Lumbricals are quite unique as they connect the flexors of the digits to the extensors and that both of their attachments are mobile. The articular system in the digits is connected by mechanical links and lumbrical muscle is one of the links of this system that produces dynamic controlled extension of interphalangeal joints [6]. Many cases of the unusual muscle belly which appears in the carpal tunnel have been reported clinically as a cause of CTS, a condition which occurs when the muscle belly compresses the median nerve. Anomalous and additional lumbrical muscle as a cause of carpal tunnel syndrome has been reported in literature [2,8,9].

Haines studied flexor muscles of the forearm and hand in the mammals and lizards; he suggested that the FDS in mammals is homologous with the intrinsic muscles of the palm, and that it shifts its origin proximally in forearm [10]. Further more Koizumi et al. mentioned that the first lumbrical muscle and the distal muscle belly for the index finger of the FDS have an intimate relationship with each other, and have a common phylogenetic origin [4]. From the above discussion it is quite clear that the additional muscle belly for the first lumbrical as observed in the present case has a phylogenetical significance and the occurrence of such an anomalous muscle belly may compress the median nerve and cause the CTS.

## Conclusion

Clinicians and hand surgeons should be aware of such a variant of first lumbrical while dealing with the hand, during various surgical procedures.

## Competing interests

The authors declare that they have no competing interests.

## Authors' contributions

SRN wrote the case report, performed the literature review and obtained written consent. RR and AK performed the literature search and assisted with writing the paper. RC obtained the photograph for the study. LVP conceived the study and helped to draft the manuscript. All authors have read and approved the final version manuscript.

## Consent

"Written informed consent was obtained from the subject's relative for publication of this case report. A copy of the written consent is available for review by the Editor-in-Chief of this journal."
